# The impact of blood transcriptomic biomarker targeted tuberculosis preventive therapy in people living with HIV: a mathematical modelling study

**DOI:** 10.1186/s12916-021-02127-w

**Published:** 2021-10-29

**Authors:** Tom Sumner, Simon C. Mendelsohn, Thomas J. Scriba, Mark Hatherill, Richard G. White

**Affiliations:** 1 TB Modelling Group, TB Centre, Centre for Mathematical Modelling of Infectious Diseases, Department of Infectious Disease Epidemiology, London School of Hygiene & Tropical Medicine, London, UK; 2 South African Tuberculosis Vaccine Initiative, Institute of Infectious Disease and Molecular Medicine, Division of Immunology, Department of Pathology, University of Cape Town, Cape Town, South Africa

**Keywords:** Tuberculosis, HIV, Preventive therapy, Biomarker, Modelling

## Abstract

**Background:**

Tuberculosis (TB) preventive therapy is recommended for all people living with HIV (PLHIV). Despite the elevated risk of TB amongst PLHIV, most of those eligible for preventive therapy would never develop TB. Tests which can identify individuals at greatest risk of disease would allow more efficient targeting of preventive therapy.

**Methods:**

We used mathematical modelling to estimate the potential impact of using a blood transcriptomic biomarker (RISK11) to target preventive therapy amongst PLHIV. We compared universal treatment to RISK11 targeted treatment and explored the effect of repeat screening of the population with RISK11.

**Results:**

Annual RISK11 screening, with preventive therapy provided to those testing positive, could avert 26% (95% CI 13–34) more cases over 10 years compared to one round of universal treatment. For the cost per case averted to be lower than universal treatment, the maximum cost of the RISK11 test was approximately 10% of the cost of preventive therapy. The benefit of RISK11 screening may be greatest amongst PLHIV on ART (compared to ART naïve individuals) due to the increased specificity of the test in this group.

**Conclusions:**

Biomarker targeted preventive therapy may be more effective than universal treatment amongst PLHIV in high incidence settings but would require repeat screening.

**Supplementary Information:**

The online version contains supplementary material available at 10.1186/s12916-021-02127-w.

## Background

Preventive therapy is a key part of tuberculosis (TB) control that has been shown to significantly reduce the individual level risk of incident TB [1, 2]. WHO guidelines [3] recommend preventive therapy for all adults and adolescents living with human immunodeficiency virus (HIV) irrespective of their degree of immunosuppression or antiretroviral therapy (ART) status. Despite these strong recommendations, uptake of preventive therapy amongst PLHIV remains limited [4].

While there is evidence that PLHIV with prior exposure to *Mycobacterium tuberculosis* (*M.tb*), as measured by tuberculin skin test (TST) or interferon gamma release assays (IGRA), may gain most benefit from preventive therapy [2], these tests have poor positive predictive value for predicting incident TB [5] and may give false negative results in immunocompromised patients. Due to this limited predictive performance and operational barriers to implementing TST and IGRA, World Health Organization (WHO) recommendations state that testing for *M.tb* infection is not required to start preventive treatment, especially in high TB incidence settings [3].

However, despite the elevated risk of TB amongst PLHIV, most of those eligible for preventive therapy would never develop TB. There is also evidence that in high TB incidence settings, the duration of protection from preventive therapy is limited [6–8]. As a result, universal short-course preventive therapy in PLHIV may not be the most effective approach to prevent TB. Strategies that could target preventive treatment to individuals at highest risk of developing TB, at the time of highest risk, may reduce TB incidence and make better use of resources.

The WHO and the Foundation for Innovative New Diagnostics (FIND) have developed a target product profile (TPP) for prognostic tests that could predict incident TB (with minimum and optimum sensitivity and specificity defined as 75% and 90% respectively) [9]. Several blood transcriptional signatures have been identified as potential predictors of development of incident disease [8, 10–13]. The performance of RISK11, an 11-gene transcriptomic host-response blood signature was recently evaluated in PLHIV in the CORTIS-HR study [14]. Amongst HIV-positive adults in South Africa, RISK11 was able to predict progression to TB in those without prevalent TB at baseline, with sensitivity of 88.6 (43.5–98.7) and specificity of 68.9 (65.3–72.3) over 15 months following screening, approaching the TPP minimum benchmarks. Because preventive therapy for PLHIV is standard of care in South Africa the trial was not able to evaluate the benefit of using RISK11 to target preventive therapy. Mathematical modelling can be used to estimate the potential effects of RISK11 targeted preventive therapy, to compare different strategies and to explore longer time horizons than are possible in clinical trials.

In this paper, we use a mathematical model, informed by data from the Correlate of Risk Targeted Intervention Study in High Risk Populations (CORTIS-HR) [14, 15], to simulate different strategies for transcriptomic targeted preventive therapy amongst PLHIV. Specifically, we explore whether repeat transcriptomic screening followed by short-course preventive therapy may reduce incidence of TB amongst PLHIV compared to universal provision of preventive therapy in PLHIV as recommend by WHO.

## Methods

### Model description

**Fig. 1:**
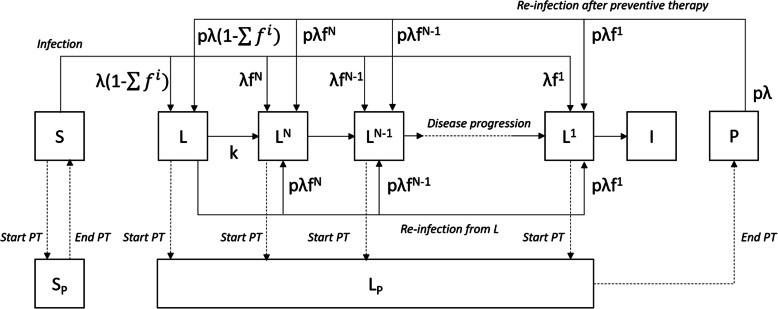
Model structure. Solid lines represent transitions between disease states; dashed lines represent transitions on and off preventive therapy. *S* = susceptible, *L* = “remote” infection, *L*^*i*^ (*i* = 1,…,*N*) = recently infected states (by time to disease), *I* = active TB disease, *S*_*p*_ = on PT (previously uninfected), *L*_*p*_ = on PT (previously infected), *P* = post PT, PT = preventive therapy, λ = risk of infection, *f*^*i*^ (*i* = 1,…,*N*) = proportion entering infect state *L*^*i*^ following infection, *p* = relative risk of infection if previously infected, *k* = risk of progression from remote infection

The model, illustrated in Fig. 1, simulates a cohort of 10,000 PLHIV over T years with no prior history of preventive therapy. The population is split into susceptible (*S*), remote infection (*L*), N recently infected states (*L*^*N*^, …*L*^*1*^), which track individuals by time until they develop disease, and those with active TB disease (*I*). This structure is used to allow us to incorporate the time horizon (15 months) of RISK11 performance.

When infected (with probability λ) susceptible individuals are assigned to one of the *L*^*i*^ (*i* = 1,..,*N*) states or *L*. The proportion entering each state (*f*^*i*^, *i* = 1,…,*N*) is based on observations of the cumulative proportion of individuals who experience disease by time since infection [16]—details of the calculation of these values are given in Additional file 1. Each of the *L*^*i*^ states is of equal duration, *D* (equal to the time step of the model), after which individuals progress to the subsequent state (*L*^*N*^ to *L*^*N*-1^ etc.) until they reach the disease state *I*. Individuals in the *L* state can progress to the *L*^*N*^ state (with probability *k*) or be re-infected at a reduced probability, *pλ* to represent the protective effect of prior infection [17]) and move to one of the *N* recent infection states in the same proportions as following initial infection. The cumulative proportion experiencing disease by time since infection is taken from data in HIV-uninfected populations and is multiplied by RR_HIV_ to represent the increased risk of TB amongst PLHIV. Details of the model parameter values and uncertainty distributions are given in Table 1.

**Table 1:** Parameter ranges used in model. 3HP, weekly isoniazid and rifapentine for 12 weeks. IPT, isoniazid preventive therapy; ART, antiretroviral therapy status

Parameter	Median (95% range)	Source
Sensitivity of IGRA for infection, se	61 (47–75)	[18]
Specificity of IGRA for infection, sp	96 (94–98)	[19]
RR of infection if previously infected, *p*	0.21 (0.14–0.30)	[17]
Duration of preventive therapy, *D*_*p*_	3 months	Based on duration of 3HP
Number of recent infection states, *N*	20	Gives total duration or recent infection of 5 years
Duration of recent infection states, *D*	3 months	
Progression from remote infection, *k*	1.5e−4	See Additional file 1
**Sensitivity for incident TB (over 15 months from testing)**		
RISK11 (whole cohort)	88.6 (43.5–98.7)	[14, 15] No incident TB cases amongst RISK11-negative participants not receiving IPT
RISK11 (on ART and not receiving IPT)	100	
RISK11 (ART naïve and not receiving IPT)	100	
Optimum TPP	90	[9]
Minimum TPP	75	
**Specificity for incident TB (over 15 months from testing)**		
RISK11 (whole cohort)	68.9 (65.3–72.3)	[14]
RISK11 (on ART and not receiving IPT)	70.2 (64.0–75.7)	
RISK11 (ART naïve and not receiving IPT)	45.9 (34.9–57.4)	
Optimum TPP	90	[9]
Minimum TPP	75	

A proportion (*C*) of the population is offered preventive therapy (*C* will depend on the strategy being considered (see below)). A proportion, *U*, of those offered treatment initiate treatment and amongst those treatment is effective in a proportion *E*. Those who are effectively treated move to on treatment states (*S*_*P*_ and *L*_*P*_ for those who were susceptible or infected respectively). While on treatment (for a duration *D*_*p*_), individuals are protected from infection or progression to disease. After treatment susceptible people return to the *S* state. People who were previously infected enter a post-treatment state *P*. These individuals are not at risk of progressing to disease but can be re-infected (at a rate *pλ*, i.e. they retain the same protection as latently infected individuals).

In our main analysis, we simulated a cohort representative of the CORTIS-HR study population, where the prevalence of IGRA positivity (*P*_IGRA_) was approximately 45%. We used the prevalence of IGRA positivity together with the estimated sensitivity (se) [18] and specificity (sp) [19] of IGRA for *M.tb* infection to estimate the true prevalence of infection (*P*_*L*_) using the following expression:

$$ {P}_L=\frac{P_{\mathrm{IGRA}}-1+ sp}{se-1+ sp} $$

We then used this estimate of the true prevalence of infection and the average age of the cohort to calculate the annual risk of infection (ARI, *λ*) (assuming a constant life-time risk of infection). The estimated prevalence of infection and ARI were then used to define the initial state of the cohort.

The relative risk of TB disease in PLHIV (*RR*_HIV_) was estimated by fitting the model to the observed incidence of TB (confirmed by positive Xpert MTB/RIF, Ultra, and/or MGIT culture on at least two separate sputum samples) over 15 months in the CORTIS-HR study: 0.9 (0.3–1.6) per 100 person years. Full details of the model fitting are given in Additional file 1. In summary, 100,000 parameter sets were sampled from the distributions given in Table 1. For each parameter set, the model was run (with no preventive therapy) to simulate the incidence of TB over 15 months, and the likelihood was calculated for each parameter set. One thousand parameter sets were then resampled with replacement from the 100,000 samples using the likelihood as weighting for the probability of selecting a given sample.

### Preventive therapy strategies

We compare a no treatment scenario to a universal preventive therapy scenario (a single round of preventive therapy offered to everyone in the cohort at *t* = 0) and to blood transcriptomic targeted therapy. In each case, we assume treatment is with isoniazid and rifapentine for 12 weeks (3HP). For the transcriptomic targeted strategies, we considered three tests: one with characteristics of the RISK11 assay (from the CORTIS-HR study) and tests that meet the minimum and optimum criteria for tests for incipient TB set out in the WHO TPP [9]. The performance characteristics of the tests are given in Table 1. For each test, we simulated a single round of screening (at *t* = 0) or repeat screening with an interval between screening of 1, 2, or 3 years. Those testing positive are offered preventive therapy. We assumed that individuals would only be offered preventive therapy once (i.e. those testing positive are not retested again irrespective of whether they initiate treatment).

We ran the model for 10 years and calculated the number of people screened, the number given preventive therapy and the number of TB cases at 5 and 10 years for each scenario. The incremental impact of test targeted preventive therapy is defined as the ratio of cases averted by test targeted preventive therapy to cases averted by universal treatment. Values greater than 1 indicate scenarios where test targeted preventive therapy averts more cases than universal treatment.

Because the likely programmatic costs of tests for incipient TB are not known we calculated the maximum cost of a test, as a proportion of the cost of a course of preventive therapy, such that (a) the total cost of the strategy with testing does not exceed the cost of the universal treatment strategy and (b) the cost per case averted of the strategy with testing does not exceed the cost per case averted of the universal treatment strategy.

The total costs (*T*) of the universal (subscript *u*) and testing strategies (subscript *t*) are:

$$ {T}_u={P}_uC $$

$$ {T}_t={P}_tC+{S}_t gC $$

where *P* = number given preventive therapy; *S* = number tested; *C* = cost of preventive therapy; and *g.C* = cost of test (defined relative to the cost of preventive therapy, *C*). Then, for the strategy with a test to cost less than universal preventive therapy:

$$ g<\frac{P_u-{P}_t}{S_t} $$

The costs per case averted (H) of the universal and testing strategies are:

$$ {H}_u=\frac{P_uC}{A_u} $$

$$ {H}_t=\frac{P_tC+{S}_t gC}{A_t} $$

where *A* = number of cases averted.

Then, for the strategy with a test to be more “cost effective” than universal preventive therapy, we need:

$$ g<\frac{1}{S_t}\left(\frac{P_u{A}_t}{A_u}-{P}_t\right) $$

### Sensitivity analysis

Uncertainty in the model parameters was explored by simulating each preventive therapy strategy for each of the 1000 parameter sets generated via the fitting process (see above). For each parameter sample, values for the RISK11 test performance were sampled from the distributions in Table 1. To identify the parameters that contribute to uncertainty in the model outputs, we calculated partial rank correlation coefficients (PRCCs) for the sampled parameters considering the ratio of cases averted and the maximum cost of the test (per case averted) as outputs.

In the CORTIS-HR study, the performance of RISK11 in PLHIV was found to vary by ART status and whether or not individuals received IPT during the study. To assess the impact of these differences on the model predictions, we carried out additional model simulations using sensitivity and specificity estimates for RISK11 based on analysis of participants who did not receive IPT during the study, stratified by ART status at enrolment in the study (see Table 1). For these sub-group analyses, *RR*_HIV_ was re-estimated by fitting the model to the observed incidence amongst either those on ART at enrolment (0.3 per 100 person-years; 95% CI 0.0–1.0) or amongst the ART naïve population (4 per 100 person-years; 95% CI 0.1–7.7). As many of those who were ART naive at enrolment initiated ART during the trial (~ 75%), reducing their risk of TB and potentially changing the performance of future RISK11 screening, we also explored a scenario in which the sensitivity and specificity of RISK11 and value of *RR*_HIV_ were varied in the model from the ART naïve cohort values to those of the ART cohort after 1 year.

In the main analysis, we assumed that uptake (*U*) was 100% and did not depend on whether testing was carried out. We also assumed that the efficacy of treatment was the same in those testing positive or negative in screening. Because we consider all outputs relative to universal treatment, they do not depend on the absolute value of treatment efficacy (*E*). Similarly, the incremental impact of test targeted treatment does not depend on uptake but the costs are proportional to the uptake.

Results from the CORTIS study [8], which evaluated the diagnostic and prognostic performance of RISK11, and the efficacy of 3HP for preventing TB in RISK11-positive, HIV-uninfected adults found that 3HP did not reduce the incidence of TB amongst RISK11-positive individuals. No data is available on the efficacy of 3HP in RISK11-positive PLHIV. To explore the possible effects of reduced efficacy of 3HP in RISK11-positive PLHIV, we carried out simulations, varying the relative efficacy of 3HP in true positive, RISK11-positive PLHIV (compared to RISK11 negative) from 0 to 100%. This reduced efficacy was also applied in the universal treatment scenario to the subset of individuals who would have been RISK11 positive.

To further explore how the results depend on the TB disease burden we re-ran the model for combinations of the prevalence of infection (0–100%) and ARI (0–5% per year).

## Results

**Fig. 2:**
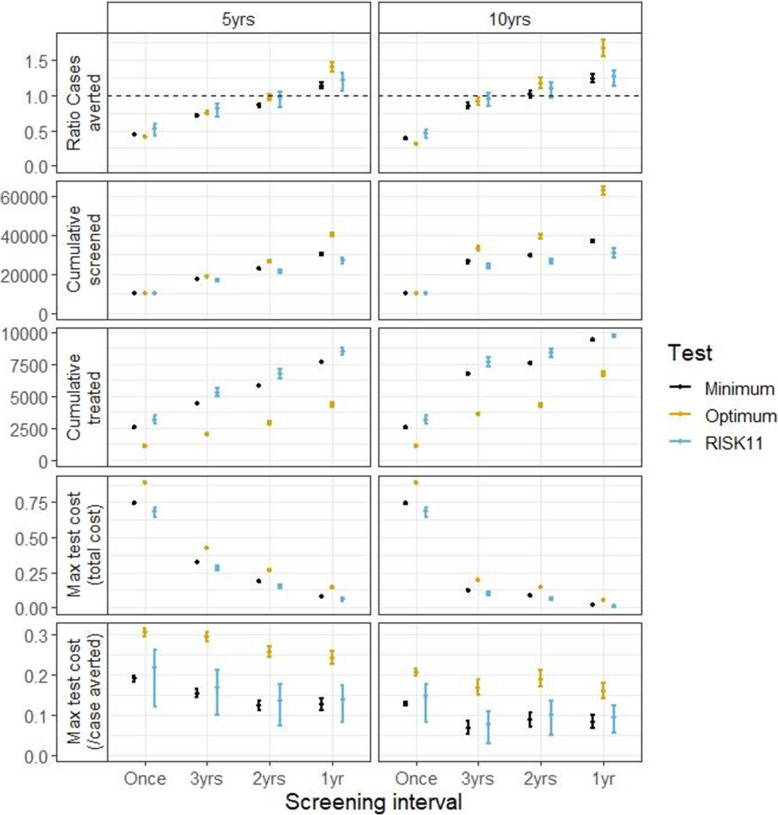
Results by test type and testing interval. Points show median model output; bars show 95% range of model outputs. Colours indicate different tests (see key). Top row: ratio of cases averted (compared to universal treatment). Second row: cumulative number screened. Third row: cumulative number given 3HP. Fourth row: maximum cost of test (relative to 3HP) such that total cost does not exceed cost of universal treatment. Fifth row: maximum cost of test (relative to 3HP) such that cost per case averted does not exceed cost per case averted of universal treatment. Left panel: after 5 years; right panel: after 10 years

Figure 2 shows the results for our main analysis of the CORTIS-HR cohort, assuming uptake of preventive therapy of 100% and equal efficacy in RISK11-positive and RISK11-negative individuals. Based on the prevalence of IGRA positivity in the trial cohort, the estimated prevalence of infection was 71% (95% CI 59–93) and the ARI was 3.6% (95% CI 2.4–7.5).

Test targeted treatment would avert at least as many cases as universal treatment with an annual screening interval (over a 5-year time horizon) or with a screening interval of 2 years or less (over a 10-year horizon). Annual RISK11 screening could avert 22% (95% CI 6–31) more cases over 5 years and 26% (95% CI 13–34) over 10 years compared to universal treatment.

With annual RISK11 screening, the test would have to cost less than 6% (95% CI 5–7) of the cost of preventive therapy (5-year horizon) or less than 1% (95% CI 0.6–1.3) of the cost of preventive therapy (10-year horizon) for the total cost of the intervention to be less than universal treatment. However, when accounting for the additional cases averted with annual RISK11 screening, the possible cost of the test is increased. The RISK11 test could cost up to 14% (95% CI 8–17) of the cost of preventive therapy (5-year horizon) or 9% (95% CI 5–12) of the cost of preventive therapy (10-year horizon) for the cost per case averted to be less than universal treatment. Calculation of partial rank correlation coefficients (PRCCs) shows that with a single round of screening, the results are most sensitive to the sensitivity and specificity of RISK11. As expected, a more sensitive and less specific test would avert more cases while a more sensitive and more specific test would result in a higher potential cost for the test. With repeat screening, the relative protective effect of prior infection is increasingly important, indicating that the risk of TB after preventive therapy is important in determining the effect of a screening based strategies. PRCCs are shown in figure Additional file 1 L: Fig S3.

Annual screening with a test satisfying the minimum TPP characteristics (75% sensitivity and specificity) prevents fewer cases than RISK11 (due to the lower sensitivity) but uses less preventive therapy (due to the higher specificity). The median cost of such a test is lower than RISK11; however, the confidence intervals overlap. A test satisfying the optimum TPP (90% sensitivity and specificity) could cost approximately 25% of the cost of preventive therapy (5-year horizon) or 16% of the cost of preventive therapy (10-year horizon) for the cost per case averted to be less than universal treatment.

**Fig. 3:**
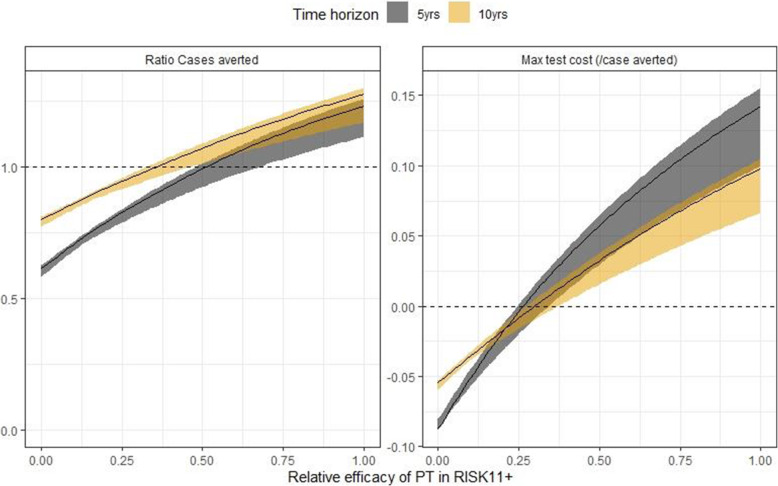
Results by relative efficacy of preventive therapy in RISK11-positive individuals. Solid lines show median model output; shaded regions show 95% range of model outputs. Colours indicate different time horizons (see key). Left panel: ratio of cases averted (compared to universal treatment). Right panel: maximum cost of test (relative to 3HP) such that cost per case averted does not exceed cost per case averted of universal treatment

As these results are calculated relative to universal treatment, they do not depend on the value of treatment efficacy (*E*) used assuming efficacy is the same by RISK11 status. If the efficacy of treatment is lower amongst RISK11 positives, the incremental benefit of RISK11 screening compared to universal treatment is lower and the test would have to cost less. Figure 3 shows how the ratio of cases averted (compared to universal treatment) and the cost of testing for annual RISK11 screening vary with the relative efficacy of 3HP in RISK11-positive PLHIV. For RISK11 targeted treatment to avert more cases over a 10-year horizon, the relative efficacy must be greater than 47%. The cost results also depend on the assumed uptake of preventive therapy. With lower uptake the number of courses of preventive therapy saved by RISK11 screening is greater and so the maximum relative cost of the test is lower; this relationship is linear, i.e. for an uptake of 75%, the cost per case averted would be 75% of that with 100% uptake.

**Fig. 4:**
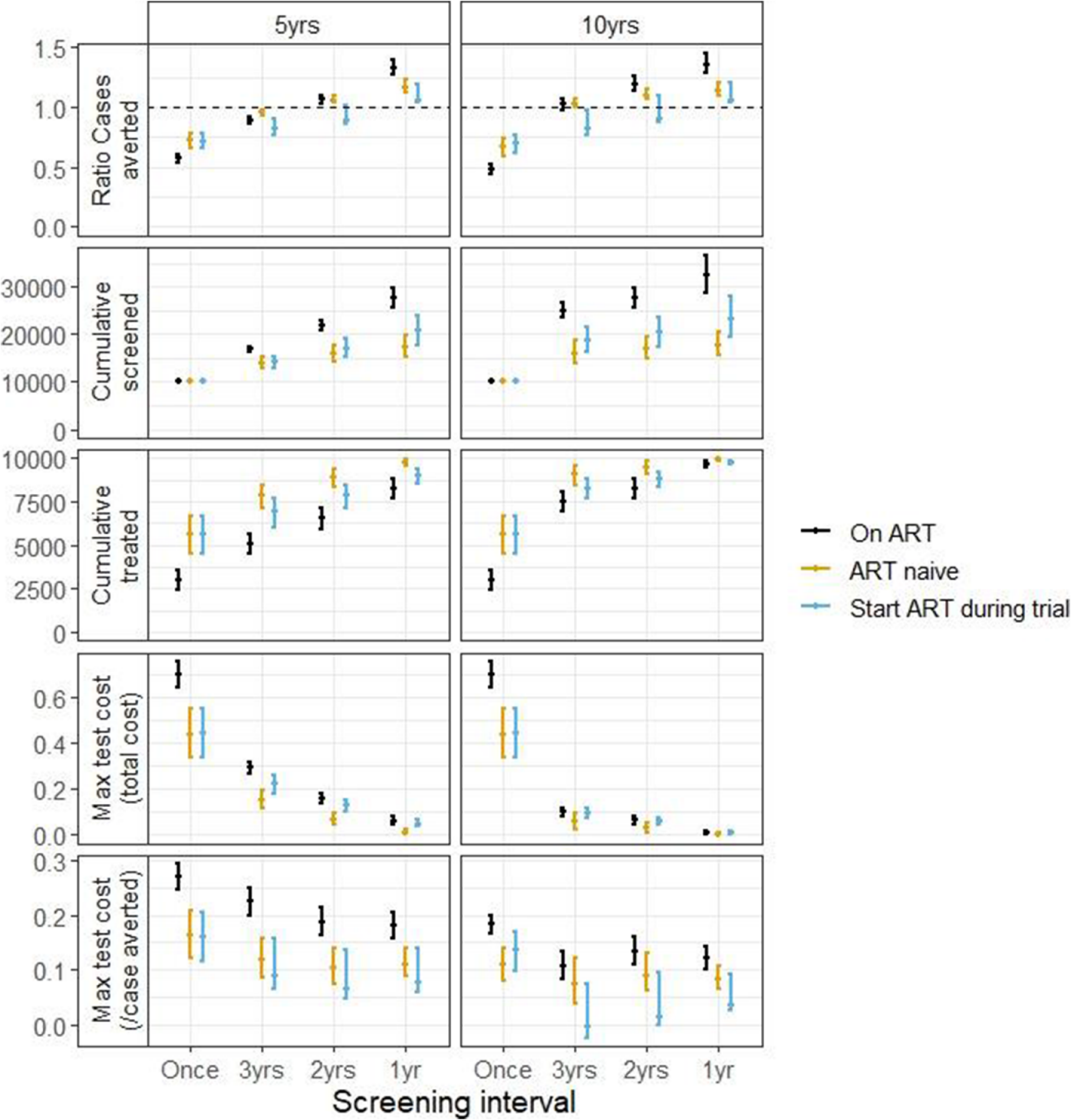
Results by ART status amongst individuals not receiving IPT during the CORTIS-HR study. Points show median model output, bars show 95% range of model outputs. Colours indicate ART status (see key). Top row: ratio of cases averted (compared to universal treatment). Second row: cumulative number screened. Third row: cumulative number given 3HP. Fourth row: maximum cost of test (relative to 3HP) such that total cost does not exceed cost of universal treatment. Fifth row: maximum cost of test (relative to 3HP) such that cost per case averted does not exceed cost per case averted of universal treatment. Left panel: after 5 years; right panel: after 10 years

Figure 4 shows the results for the sub-group analysis of those who did not receive IPT during the CORTIS-HR study stratified by ART status at enrolment. Annual RISK11 screening is more effective than universal treatment in all sub-groups. However, the incremental benefit of annual RISK11 screening (compared to universal treatment) is greater in those on ART than in ART naïve individuals and uses less preventive therapy (due to the higher specificity of RISK11 in people on ART). As a result, the test could cost more (relative to the cost of preventive therapy) when used in individuals on ART (12% (95% CI 10–14)).

**Fig. 5:**
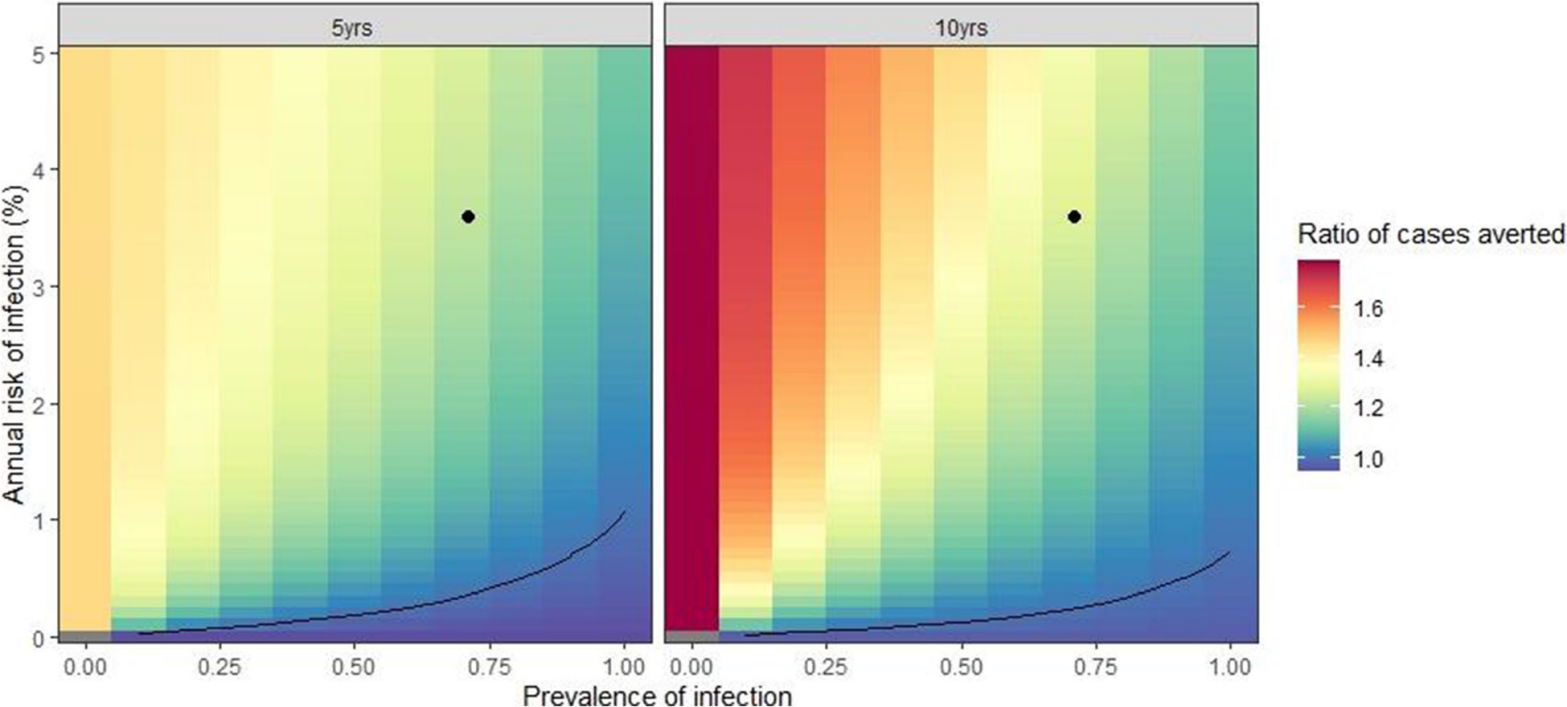
Ratio of cases averted by annual RISK11 targeted preventive therapy compared to universal treatment by prevalence of infection (*x*-axis) and annual risk of infection (*y*-axis). Left panel: after 5 years; right panel: after 10 years. Black lines show the contour where the ratio = 1. Black dots indicate the median prevalence and ARI assumed in the CORTIS-HR cohort (Fig. 2)

**Fig. 6:**
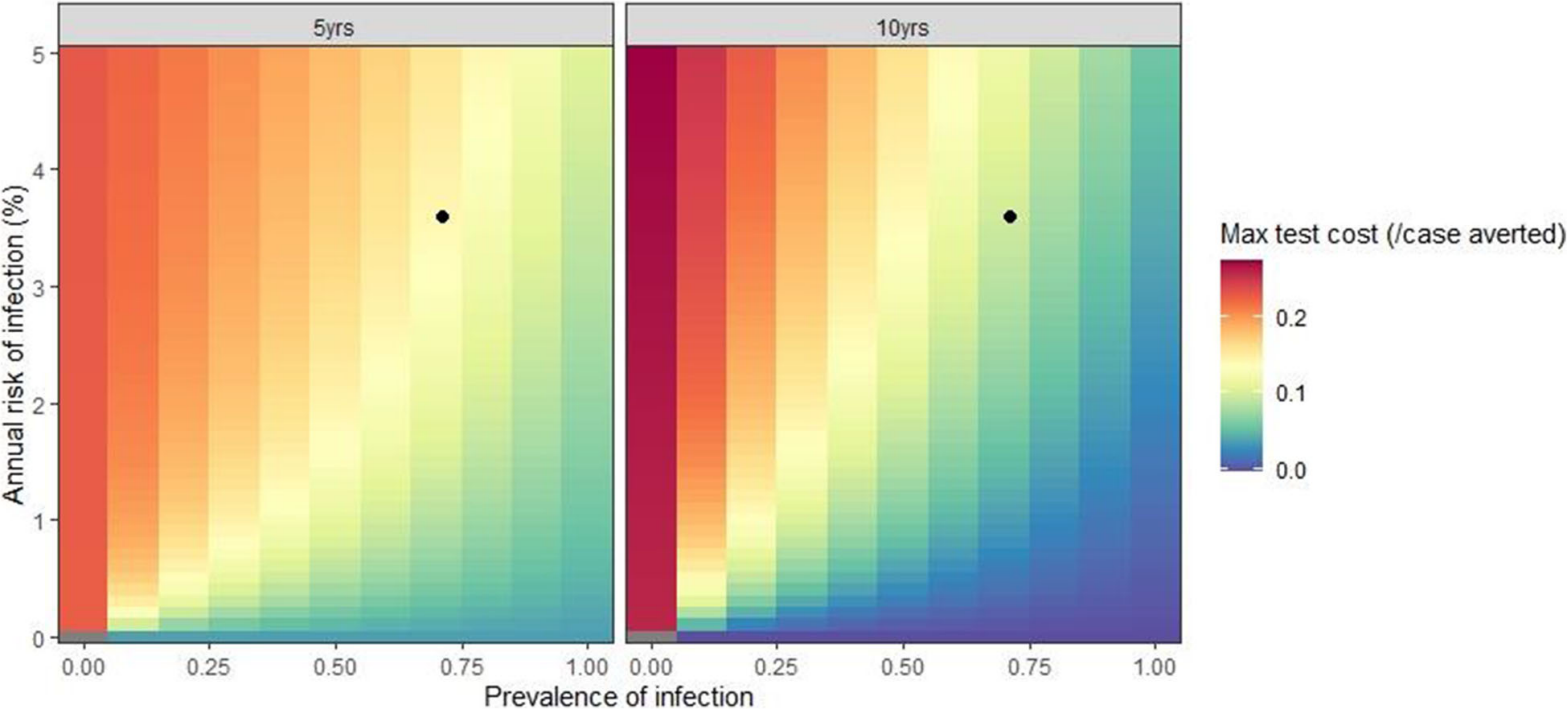
Maximum cost of RISK11 test (relative to 3HP) such that the cost per case averted does not exceed the cost per case averted of universal treatment by prevalence of infection (*x*-axis) and annual risk of infection (*y*-axis). Left panel: after 5 years; right panel: after 10 years. Black dots indicate the median prevalence and ARI assumed in the CORTIS-HR cohort (Fig. 2)

Figure 5 shows the ratio of cases averted by annual RISK11 targeted preventive therapy compared to universal treatment, for different values of prevalence of infection and ARI. Figure 6 shows the maximum cost of the RISK11 test (per case averted). The lower the prevalence of infection the greater the incremental benefit of RISK11 targeted preventive therapy and the more the test can cost. This is because, at a low prevalence, there are fewer infected individuals who benefit from universal preventive therapy. Conversely, the higher the ARI the more additional cases are averted by RISK11 targeted screening and the more the test can cost. This is because, at a higher ARI, more individuals would be infected after universal treatment and there is greater benefit from using RISK11 to target treatment at a later time.

## Discussion

Our results suggest that targeting short-course preventive therapy in PLHIV based on repeat transcriptomic screening could prevent more incident TB cases than a single round of universal treatment as recommended by WHO. Current WHO guidelines recommend that all PLHIV should be offered preventive therapy and that testing for M.tb infection is not a pre-requisite for initiating treatment. National guidelines vary, but in South Africa, the location of the CORTIS-HR study, the recommendation is also that all PLHIV are eligible for treatment with the duration of therapy dependent on TST status. In a high TB incidence setting, such as South Africa, screening would need to be conducted at least biennially to be more effective than universal treatment over 10 years. The use of transcriptomic testing would also require fewer people to be treated with preventive therapy—the total number treated only approaches the total population with annual screening over a 10-year time horizon. Given the extra cases averted and reduction in the number treated, the cost of testing could be approximately 10% of the cost of preventive therapy for annual screening to be more cost-effective (cost per case averted) compared to universal treatment. Assuming a full cost of 3HP of approximately 70 USD in South Africa [20], the cost of testing could be on the order of 7 USD. For comparison, the cost of a prognostic test defined in the TPP (minimal characteristics) was 10–100 USD [9].

Our results do not depend on the absolute level of efficacy of preventive therapy assuming treatment is equally efficacious in RISK11-positive and RISK11-negative individuals. However, if treatment is less effective in RISK11-positive individuals, the benefit of targeted preventive therapy is reduced; with a relative efficacy of below 47%, annual RISK11 screening becomes less effective than universal treatment. The CORTIS study [8], which evaluated the diagnostic and prognostic performance of RISK11 in HIV-uninfected adults, found that RISK11 targeted preventive therapy with 3HP did not reduce the risk of incident TB through 15 months follow up; however, there were no incident TB cases through 9 months in adherent RISK11-positive participants who completed at least 11 out of 12 observed doses of once-weekly isoniazid-rifapentine within 16 weeks suggesting a short term benefit of 3HP in this population. No data is available on the efficacy of treatment in RISK11-positive, HIV-infected populations.

In our analysis, we have assumed that uptake and completion of preventive therapy does not depend on testing. It is possible that uptake and completion may be influenced by testing. For example, data from the US suggests that individuals tested with IGRA had increased rates of preventive therapy completion compared to those tested with TST [21]. However, it is also possible that testing may reduce uptake if there is a delay between testing and availability of test results.

We found that the additional benefit of RISK11 targeted treatment and the potential cost of the test depend on the prevalence of infection and the annual risk of infection. The model suggests that for an ARI above 1%, annual screening with RISK11 would avert more cases of TB than universal treatment for any prevalence of infection. The maximum relative cost for a test, across the range of ARI and prevalence considered, was 27% of the cost of a course of preventive therapy, providing a potential upper bound on the costs.

Previous studies have compared the cost-effectiveness of TST or IGRA targeted preventive therapy to untargeted treatment in PLHIV [22–24] but have not considered longer term dynamics or repeat screening. Data from Hong Kong showed limited benefit of repeat testing for infection amongst PLHIV [25] potentially due to the declining risk of TB over time associated with ART driven immune-recovery. We have assumed the risk of developing TB remained constant over time which may overestimate the benefit of repeat RISK11 screening. However, this study was also conducted in a low-incidence community setting where the risk of infection after initial testing is low. This would also limit any benefit of repeat testing, consistent with our result that the additional benefit of annual RISK11 screening would be lower at lower ARIs.

We assumed that individuals would only receive a single course of preventive therapy as there is limited data on the effect of repeated courses of preventive treatment. A trial in South Africa, Ethiopia, and Mozambique found no additional benefit of universal annual 3HP (compared to a single course of treatment) amongst PLHIV on ART [26]. However, targeted annual preventive therapy to those at highest risk of progression to TB disease (ART naïve, low CD4) has not been explored.

We have only considered the direct effects of preventive therapy and not the indirect benefit of reductions in TB incidence on onward transmission. We only considered the costs saved from reduced use of preventive therapy when calculating the maximum relative costs of testing. However, there are potential additional cost savings of annual RISK11 screening due to the increased number of TB cases averted and the resulting reduction in costs of treating active TB. Finally, we do not consider the potential benefit of identifying undiagnosed prevalent TB during RISK11 screening. Guidelines recommend the use of symptom screening to exclude prevalent disease before initiation of preventive therapy [3]; however, data from the CORTIS-HR study found that symptom screening missed approximately 70% of prevalent cases while RISK11 had sensitivity of 87.5 (58.3–100) for undiagnosed prevalent disease.

## Conclusion

Biomarker targeted preventive therapy may be more effective than one-off of universal treatment amongst PLHIV in high incidence settings but would require repeat screening. Assuming annual screening, testing would have to cost on the order of one tenth the cost of the preventive therapy regimen to be more cost-effective than one-off universal treatment.

## Supplementary Information

**Additional file 1.** Additional details of model, results of model fitting and results of parameter sensitivity analysis. **Figure S1.** Model structure. **Figure S2.** Cumulative incidence of TB by time since infection used to calculate the model parameters. **Table S1.** Parameters used to distribute new infections to recent latent states. **Table S2.** Prior parameter distributions used in model fitting. **Table S3.** Observed and fitted TB incidence (per 100 person years). **Table S4.** Posterior parameter ranges by sub-group**. Figure S3.** Partial rank correlation coefficients for the model parameters.

## Data Availability

All code used in the study is available at: https://github.com/tomsumner/CORTIS_HR_CE

## Abbreviations

TB: Tuberculosis
HIV: Human immunodeficiency virus
ART: Antiretroviral therapy
PLHIV: Human immunodeficiency virus
M.tb: Mycobacterium tuberculosis
TST: Tuberculin skin test
IGRA: Interferon gamma release assay
WHO: World Health Organization
FIND: Foundation for Innovative New Diagnostics
TPP: Target product profile
CORTIS-HR: Correlate of Risk Targeted Intervention Study in High Risk Populations
ARI: Annual risk of infection
PRCCs: Partial rank correlation co-efficient
3HP: Weekly isoniazid and rifapentine for 12 weeks
