# Supplementary material for: The impact of blood transcriptomic biomarker targeted tuberculosis preventive therapy in people living with HIV: a mathematical modelling study
Source: BMC Med. 2021 Oct 29;19:252. doi: 10.1186/s12916-021-02127-w (PMC8555196; doi:10.1186/s12916-021-02127-w)
Supplement: Supplementary file 1 — Additional file 1. Additional details of model, results of model fitting and results of parameter sensitivity analysis. Figure S1. Model structure. Figure S2. Cumulative incidence of TB by time since infection used to calculate the model parameters. Table S1. Parameters used to distribute new infections to recent latent states. Table S2. Prior parameter distributions used in model fitting. Table S3. Observed and fitted TB incidence (per 100 person years). Table S4. Posterior parameter ranges by sub-group. Figure S3. Partial rank correlation coefficients for the model parameters. [file 12916_2021_2127_MOESM1_ESM.docx]

**Additional File 1**

The impact of blood transcriptomic biomarker targeted tuberculosis preventive therapy in people living with HIV: a mathematical modelling study.

Tom Sumner^1*^, Simon C Mendelsohn^2^, Thomas J Scriba^2^, Mark Hatherill^2^, Richard G White^1^

^1^TB Modelling Group, TB Centre, Centre for Mathematical Modelling of Infectious Diseases, Department of Infectious Disease Epidemiology, London School of Hygiene & Tropical Medicine, London, United Kingdom.

^2^South African Tuberculosis Vaccine Initiative, Institute of Infectious Disease and Molecular Medicine, Division of Immunology, Department of Pathology, University of Cape Town, South Africa.

**Additional details of model structure**

The general structure of the model (Figure S1 (reproduces figure 1 from main text)) is described in the main text.

Here we present additional details on the distribution of infections to the L^i^ states and the process of implementing the model.


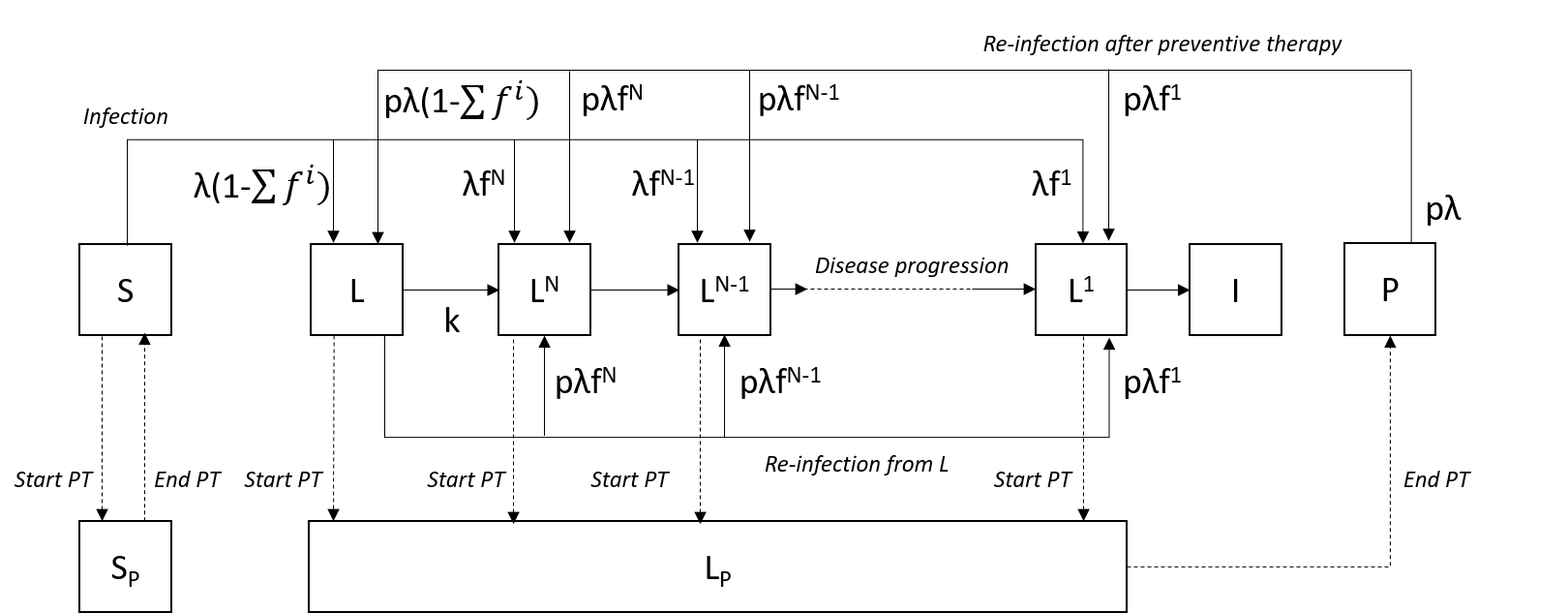


**Figure S1. Model structure.** Solid lines represent transitions between disease states, dashed lines represent transitions on and off preventive therapy. S = susceptible, L = “remote” infection, L^i^ (i=1,…,N) = recently infected states (by time to disease), I = active TB disease, S_p_ = on PT (previously uninfected), L_p_ = on PT (previously infected), P = post PT, PT = preventive therapy, λ = risk of infection, f^i^ (i=1,…,N) = proportion entering infect state L^i^ following infection, p = relative risk of infection if previously infected, k = risk of progression from remote infection.

The model is implemented using a transition matrix that determines the probability of moving between states in a time step. In each time step the status of the population is updated by multiplying the current population state by the transition matrix. The matrix is assumed to be constant, except in time steps where screening is conducted – here the rates of transition from the susceptible and infected states to the on preventive therapy states are changed from zero (default values) to values determined by the sensitivity and specificity of the screening test (RISK11 or minimum or optimal TPP), the uptake of preventive therapy and the efficacy of preventive therapy. The model assumes individuals who screen positive are not screened again (i.e. if they are only eligible for treatment once) – this is implemented by duplicating the susceptible and infected states to stratify by eligibility for preventive therapy (for simplicity this is not shown in figure S1). The time step of the model is taken as 3 months to match the duration of preventive therapy (3HP).

The proportion of individuals entering each latent state (f^i^) is calculated from observations of the cumulative incidence of TB among recently exposed individuals. These observations come from the control arm of BCG trials conducted in the UK in the 1950s [16]. Figure S2 shows the cumulative incidence by year since infection. To calculate the proportion entering each infection state we first calculate the difference between cumulative incidence in sequential years. Because we use a 3-month time step in the model we then divide this proportion by 4 – this assumes the incidence of TB in a given year is equally divided across the year. The resulting values of f^i^ are given in table S1. The remainder of those infected enter the remote infection state, L. The rate of progression from L to the first recent infection state L^N^ is calculated to match the long term cumulative incidence of TB (measured over 10 years of follow up) of 11%.

We assume that these values are constant, such that the shape of the cumulative incidence curve does not change. However, the absolute risk of disease is derived by multiplying these values by the relative risk in PLHIV which is assumed to be uncertain (see section on model fitting below).


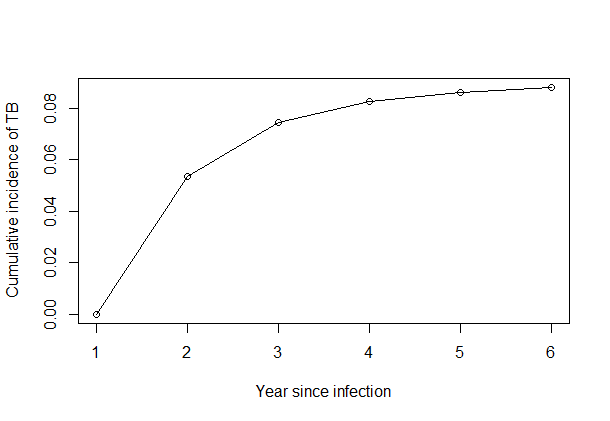


**Figure S2.** Cumulative incidence TB by time since infection used to calculate the model parameters.

| **Infection state, L^i^** | **Proportion entering state, f^i^** |
| --- | --- |
| 1 - 4 | 1.34% |
| 5-8 | 0.52% |
| 9-12 | 0.21% |
| 13-16 | 0.08% |
| 17-20 | 0.04% |

**Table S1. Parameters used to distribute new infections to recent latent states.**

**Additional details and results of model fitting**

The model is fitted to the observed incidence of infection in the CORTIS-HR cohort by varying the relative risk of TB in PLHIV (RR_HIV_). The model is fitted using a sampling-importance-resampling approach. 100,000 parameter sets are generated from the parameter distributions shown in table S2. For each parameter set the model is simulated to 15 months (the duration of the CORTIS-HR trial), the modelled incidence of TB is calculated and the likelihood of the parameter set calculated. We assume that the observed incidence is log-normally distributed. 1000 parameter sets are then sampled (with replacement) using the likelihood as the probability of selecting any given sample.

This process was repeated for the whole cohort and for each sub-group (on ART, no IPT; ART naïve, no IPT) included in the secondary analysis described in the main text. Table S3 shows the model and observed incidence after fitting. Table A4 shows the resulting distributions of the model parameters by sub-group. Posterior ranges for all parameters, except RR_HIV_, were not significantly different from the prior distributions and did not differ by sub-group.

The 1000 parameter sets were then used to simulate the preventive therapy strategies. For each parameter set the values of the RISK11 performance (sensitivity and specificity) were sampled from the ranges given in table 1 in the main text.

| **Parameter** | **Median (95% range)** | **Distribution** |
| --- | --- | --- |
| Sensitivity of IGRA for infection, se | 61 (47-75) | Beta(22.20,14.354) |
| Specificity of IGRA for infection, sp | 96 (94-98) | Beta(420.99,17.84) |
| RR of infection if previously infected, p | 0.21 (0.14-0.30) | Beta(21.32,79.25) |
| Relative risk of TB in PLHIV, RR_HIV_ |  | Uniform(1,50) |

**Table S2. Prior parameter distributions used in model fitting**

| **Sub-group** | **Observed incidence** | **Model incidence** |
| --- | --- | --- |
| Whole cohort | 0.3 (0.9-1.6) | 0.25 (0.98-1.94) |
| On ART and not receiving IPT | 0 (0.3-1) | 0.19 (0.35-0.66) |
| ART naïve and not receiving IPT | 4 (0.1-7.7) | 4.6 (0.44-9.44) |

**Table S3. Observed and fitted TB incidence (per 100 person years)**

| **Parameter** | **Whole cohort** | **On ART and not receiving IPT** | **ART naïve and not receiving IPT** |
| --- | --- | --- | --- |
| Sensitivity of IGRA for infection, se | 0.62 (0.48-0.75) | 0.63 (0.49-0.77) | 0.61 (0.48-0.76) |
| Specificity of IGRA for infection, sp | 0.96 (0.94-0.98) | 0.96 (0.94-0.97) | 0.96 (0.94-0.98) |
| RR of infection if previously infected, p | 0.21 (0.14-0.29) | 0.21 (0.14-0.29) | 0.21 (0.14-0.30) |
| Relative risk of TB in PLHIV, RR_HIV_ | 5.3 (1.3-11.2) | 2.0 (1.1-3.8) | 25.2 (2.2-48.5) |

**Table S4. Posterior parameter ranges by sub-group**

**Parameter sensitivity analysis results**


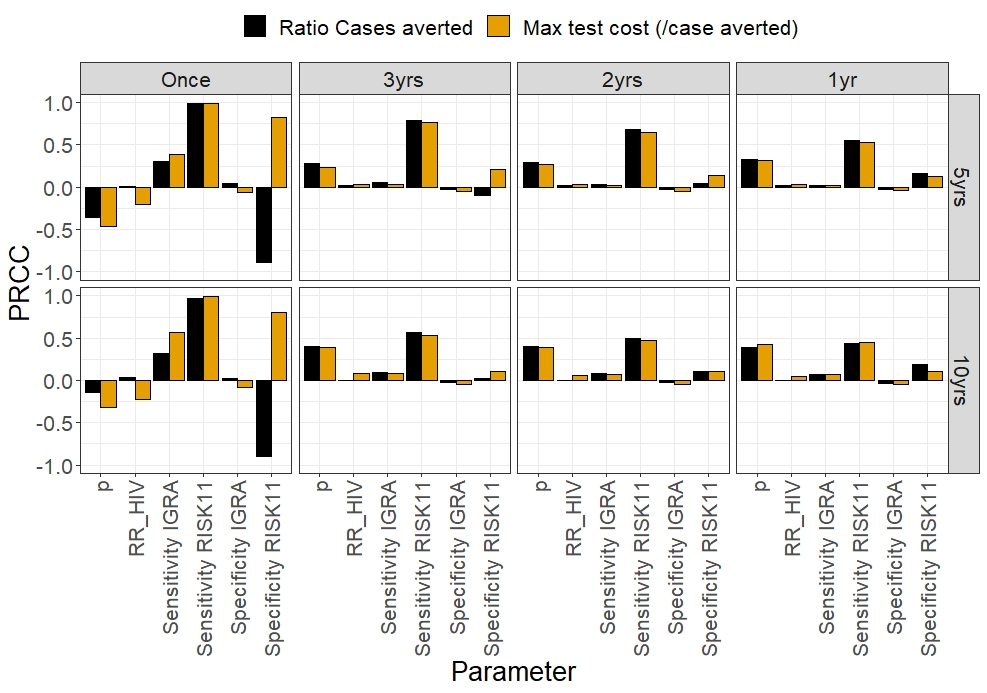


**Figure S3.** Partial rank correlation coefficients for the model parameters. Panels show results by frequency of RISK11 screening, rows by time horizon. Black bars show values using the ratio of cases averted (compared to universal treatment), orange bars show the maximum cost of the test (relative to 3HP) such that the cost per test averted is less than universal treatment.
